# Risk factors for interstitial lung disease induced by gemcitabine plus albumin-bound paclitaxel therapy in pancreatic ductal adenocarcinoma patients

**DOI:** 10.1186/s40780-021-00236-5

**Published:** 2022-02-02

**Authors:** Rikako Ueda, Naho Yamamoto, Yuki Hori, Kouji Yoshida, Koushiro Ohtsubo, Takeshi Terashima, Tsutomu Shimada, Yoshimichi Sai

**Affiliations:** 1 Department of Hospital Pharmacy, University Hospital, Kanazawa University, 13-1 Takaramachi, Kanazawa, Ishikawa Japan; 2 Division of Medical Oncology, Cancer Research Institute, Kanazawa University, 13-1 Takaramachi, Kanazawa, Ishikawa Japan; 3 Department of Gastroenterology, Graduate School of Medicine, Kanazawa University, 13-1 Takaramachi, Kanazawa, Ishikawa Japan

**Keywords:** Pancreatic ductal adenocarcinoma, Interstitial lung disease, Gemcitabine plus nab-paclitaxel therapy, Risk factor, Goshajinkigan

## Abstract

**Background:**

Gemcitabine plus nab-paclitaxel (GnP) therapy is used for unresectable pancreatic ductal adenocarcinoma, but may cause interstitial lung disease (ILD) as a serious side effect. However, the risk factors for ILD in patients receiving GnP therapy are not well established. Here, we retrospectively investigated the incidence of GnP-induced ILD in pancreatic ductal adenocarcinoma patients, and the risk factors.

**Methods:**

We investigated the patients’ background, laboratory data, previous treatment history, concomitant medications, number of doses of GnP, cumulative dosage and administration period, and occurrence of side effects.

**Results:**

Of the 105 patients included in this study, ILD occurred in 10 (9.5%). Patients with ILD had a significantly higher frequency of concomitant treatment with Kampo medicines, especially goshajinkigan, which is considered to help prevent chemotherapy-induced peripheral neuropathy (CIPN) (odds ratio: 11.5, 95% confidence interval: 2.67–49.38). No significant differences were observed in other clinical characteristics. Notably, the severity of CIPN in patients who used goshajinkigan for prevention was not significantly different from that in patients who did not use goshajinkigan in this study.

**Conclusions:**

These results suggest that administration of goshajinkigan to patients receiving GnP therapy for prevention of CIPN may need to be reconsidered.

## Introduction

Gemcitabine (GEM) plus nab-paclitaxel (nabPTX) (GnP) therapy has been widely used in Japan as chemotherapy for unresectable metastatic pancreatic ductal adenocarcinoma (PDAC) since it was approved in December 2014. In the Clinical Practice Guideline for Pancreatic Cancer 2019, GnP therapy is recommended as a first-line treatment along with FOLFIRINOX therapy (oxaliplatin + irinotecan + fluorouracil + levofolinate calcium) for locally advanced unresectable or metastatic PDAC [1].

However, interstitial lung disease (ILD) is a serious side effect of many anticancer agents, including GEM, nabPTX, and especially GnP [2], and is associated with high mortality. In a phase III international multicenter study in PDAC patients, the incidence of ILD was 1% in the GEM group and 4% in the nabPTX combination group [3]. According to the Japan Respiratory Society’s Guide for Diagnosis and Treatment of Drug-induced Pulmonary Disorders, non-specific risk factors for ILD include age 60 years or older, presence of existing lung disease (particularly interstitial pneumonia, pulmonary fibrosis), post-pulmonary surgery, deterioration of respiratory function, high-concentration oxygen administration, lung irradiation, multidrug therapy with anticancer drugs, heart disease, and renal disorders [4]. Specific risk factors for GEM-related ILD include elderly status, smoking, history of chemotherapy, advanced cancer stage, history of lung disease or lung cancer, and prior thoracic radiotherapy [5–7]. In addition, poor performance status has been reported as a risk factor for ILD in patients receiving several anticancer drugs [8, 9]. As for GnP therapy, Takeda et al. reported that ABO blood type B was an independent risk factor for ILD in PDAC patients [10]. Irie et al. found that the median age of GnP-treated patients with ILD was significantly higher than that of GnP-treated patients without ILD [11]. However, the risk factors for ILD in patients receiving GnP therapy have not yet been thoroughly investigated.

Therefore, we aimed to clarify the incidence of ILD in PDAC patients receiving GnP therapy, and to investigate the risk factors, in order to provide safer chemotherapy.

## Materials & methods

### Patients

The subjects were patients who underwent GnP therapy for locally advanced unresectable or metastatic PDAC at Kanazawa University Hospital from February 2015 to March 2019. Patients who received GnP only on day 1 were excluded. A standard 4-week course consisted of nab-PTX 125 mg/m^2^, GEM 1000 mg/m^2^ administered on days 1, 8 and 15, with a drug holiday on day 22.

### Subjects for analysis

From the electronic medical records system, we collected data on pretreatment clinical characteristics, including age, gender, performance status (Eastern Cooperative Oncology Group), chemotherapy history, putative risk factors for ILD (history of lung disease, lung metastasis, lung surgery, thoracic radiotherapy, smoking, heart disease, and renal function), concomitant medications at the initiation of GnP treatment (non-steroidal anti-inflammatory agents (NSAIDs), proton pump inhibitors (PPIs), Kampo medicines, and pregabalin), administration period of concomitant medications, number of GnP doses, cumulative dosage and administration period, severity and time of onset of ILD, KL-6 level at the onset, clinical outcome, and the severity of chemotherapy-induced peripheral neuropathy (CIPN). Regarding concomitant drugs, we targeted drugs for which ILD is documented as a serious side effect, or that have been reported to cause ILD, and that were regularly used for 1 week or more, since onset of ILD is reported to occur at 1 to 6 weeks after the start of drug administration [4].

### Evaluation method

The severity of ILD and CIPN was evaluated based on the National Cancer Institute Common Terminology Criteria for Adverse Events (CTCAE) ver.5.0. ILD was diagnosed based on a comprehensive evaluation of the patients’ clinical course, physical findings (cough, respiratory distress, fever, and decreased SpO2), X-ray findings, computed tomography findings (CT) and laboratory data (serum lactate dehydrogenase, C-reactive protein, KL-6, or surfactant proteins A or D). For differential diagnosis, absence of infection was confirmed by sputum and blood culture and serodiagnostic tests (1,3-β-d-glucan, cytomegalovirus antigenemia, or procalcitonin), or lack of response to antibiotics, in order to rule out infection or other pneumonia. Treatment of ILD was determined in consultation with a pulmonologist. The severity of CIPN was evaluated based on the medical records of physicians, nurses, and pharmacists at the time of discontinuation of GnP therapy or at the time of cutoff. If CIPN had developed before the initiation of GnP therapy, the onset of CIPN was defined as the time when CIPN worsened after the initiation of GnP therapy.

### Statistical analysis

To examine risk factors, univariate analysis was performed for each factor using the Mann-Whitney *U*-test or Fisher’s exact test. *P* < 0.05 was considered statistically significant. The analysis was performed using SPSS ver. 24 statistical software (SPSS Co., Ltd., Tokyo).

### Ethics

This study was conducted with the approval of the Kanazawa University Medical Ethics Review Committee in compliance with the “Ethical Guidelines for Medical Research for Humans” (Clinical Trial No. 2018–151).

## Results

### Patients’ characteristics

One hundred and seven patients who were treated with GnP therapy were registered. At the time of the data cutoff, 10 patients were still on treatment. Two patients were excluded because they were clinically diagnosed with progressive disease (PD) after a single dose, so the therapy was discontinued. Of the remaining 105 patients, 71 were males and 34 were females. The median age was 65 years. The number of patients receiving first-line treatment was 56, and the number receiving second-line or later treatment was 49. Prior treatment included modified FOLFIRINOX, S-1, GEM, and erlotinib. The median duration of treatment was 101 days and the median number of doses was 8 (Table 1).

**Table 1:** Patients’ characteristics

	*n* = 105
Age (years)	65 (37–79)^a^
Gender (male/female), n (%)	71 (68%) / 34 (32%)
Performance status 0, n (%)	38 (36%)
1, n (%)	63 (60%)
2, n (%)	4 (4%)
Line of treatment 1st line, n (%)	56 (53%)
2nd line or later, n (%)	49 (47%)
Presence of lung disease, n (%)	9 (9%)
Presence of lung metastasis, n (%)	12 (11%)
History of pulmonary surgery, n (%)	0 (0%)
History of lung irradiation, n (%)	3 (3%)
Smoker, n (%)	55 (52%)
Presence of heart disease, n (%)	20 (19%)
GnP number of doses (time)	8 (2–63)^a^
GnP administration period (days)	101 (7–931)^a^
GEM cumulative dosage (mg)	12,050 (2400-88,200+)^a^
nabPTX cumulative dosage (mg)	1520 (360–7364+)^a^
ILD frequency, n (%)	10 (9.5%)
Period from initiation of treatment to onset (days)	79 (34–202)^a^
Cumulative number of doses of GnP (time)	8 (3–28)^a^

GEM and nabPTX cumulative dosage include patients whose treatment is ongoing

*GnP* Gemcitabine (GEM) + nab-paclitaxel (nab-PTX) therapy, *ILD* Interstitial lung disease

^a^Median (minimum-maximum)

### Concomitant medications at the initiation of treatment

Concomitant medications at the initiation of GnP therapy were PPIs in 80 patients (76%), NSAIDs in 31 patients (30%), Kampo medicines in 30 patients (29%), and pregabalin in 8 patients (8%). Some patients were using multiple drugs. Among the Kampo medicines, goshajinkigan (GJG), prescribed to prevent exacerbation of CIPN, was the most common (23 people, 22%) (Table 2).

**Table 2:** Concomitant medications at the initiation of treatment

	Patients	Percentage of all cases (%)
PPI		
Rabeprazole	38	36
Esomeprazole	23	22
Vonoprazan	15	14
Omeprazole	4	4
NSAIDs		
Loxoprofen	17	16
Celecoxib	7	7
Naproxen	5	5
Meloxicam	1	1
Tiaramide	1	1
Kampo medicines		
Goshajinkigan	23	22
Daikentyuto	3	3
Rikkunshito	2	2
Hangeshashinto	2	2
Ninjinyoeito	1	1
Juzentaihoto	1	1
Kakkonto	1	1
Bukuryoingohangeshashinto	1	1
Others		
Pregabalin	8	8

*PPI* Proton pump inhibitor, *NSAIDs* Non-steroidal anti-inflammatory drugs

### Frequency of occurrence of ILD

**Fig. 1:**
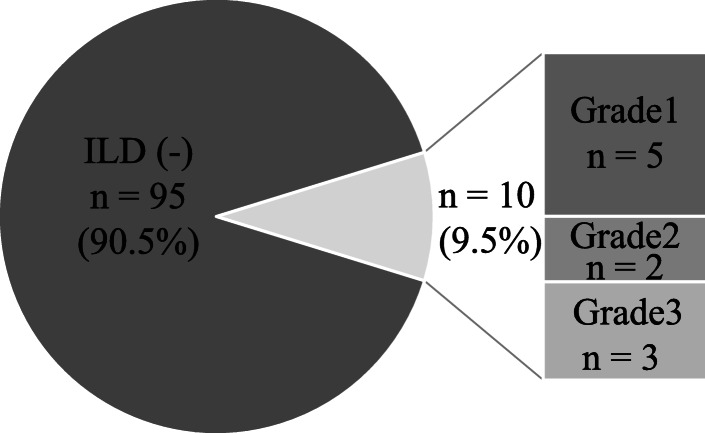
Frequency and grade of interstitial lung disease in patients receiving gemcitabine plus albumin-bound paclitaxel therapy in this study

Of the 105 patients who received GnP, 10 (9.5%) developed ILD, with 5 Grade 1 (4.8%), 2 Grade 2 (1.9%), and 3 Grade 3 or higher (2.9%). The median period from initiation to onset was 79 days and the median number of cumulative doses was 8. After the ILD onset, GnP therapy was discontinued in all cases, and the symptom improved in response to steroid therapy in all patients with Grade 2 or more, and without treatment in the Grade 1 patients (Table 1, Fig. 1).

### Risk factors for developing ILD

To identify risk factors related to GnP-induced ILD, univariate analysis was performed using patient background factors and the presence or absence of ILD. GnP-induced ILD was significantly associated with the use of Kampo medicines, especially GJG (odds ratio: 11.5, 95% confidence interval (CI): 2.67–49.38). Other patient background factors were not significantly associated with the development of ILD (Table 3).

**Table 3:** Risk factors for developing ILD

	ILD (+) n = 10	ILD (−) *n* = 95	*P*	Odds ratio	95% CI
Age (years)	70 (57–79)^c^	66 (37–79)^c^	0.127^a^		
Gender (male/female)	5 / 5	66 / 29	0.287^b^		
GnP number of doses (time)	8 (3–28)^c^	9 (2–63)^c^	0.562^a^		
GnP administration period (days)	67 (16–289)^c^	108 (7–931)^c^	0.342^a^		
Performance status 0, n (%)	1 (10%)	37 (39%)	0.129^a^		
1, n (%)	9 (90%)	54 (57%)			
2, n (%)	0 (0%)	4 (4%)			
History of chemotherapy (+/−)	4 / 6	45 / 50	0.748^b^		
History of lung disease (+/−)	1 / 9	8 / 87	1.000^b^		
Lung metastasis (+/−)	1 / 9	11 / 84	1.000^b^		
Lung irradiation (+/−)	0 / 10	3 / 92	1.000^b^		
Smoker (+/−)	4 / 6	51 / 44	0.513^b^		
Heart disease (+/−)	2 / 8	18 / 77	1.000^b^		
Scr (mg/dL)	0.62 (0.47–0.61)^c^	0.61 (0.33–1.38)^c^	0.823^a^		
NSAIDs (+/−)	3 / 7	28 / 67	1.000^b^		
PPI (+/−)	9 / 1	71 / 24	0.445^b^		
Kampo medicines (+/−)	7 / 3	23 / 72	0.005^b^		
Goshajinkigan (+/−)	7 / 3	16 / 79	0.001^b^	11.5	2.67–49.38
Pregabalin (+/−)	2 / 8	6 / 89	0.168^b^		

*GnP* Gemcitabine (GEM) + nab-paclitaxel (nab-PTX) therapy, *Scr* Serum creatinine, *NSAIDs* Non-steroidal anti-inflammatory drugs, *PPI* Proton pump inhibitor, *CI* Confidence interval

^a^Mann-Whitney *U*-test, ^b^Fisher’s exact test

^c^Median (minimum-maximum)

### Demographics of patients with ILD

Demographics of the 10 patients who developed ILD are summarized in Table 4. Age was 60 years or older except for one patient, and there was no consistency in the number of GnP administrations and the date of onset. One of the 10 cases had a history of lung disease. In addition, 4 cases had a history of treatment with modified FOLFIRINOX as the first-line chemotherapy. Six cases showed increased KL-6 (> 500 U / mL) at the onset. Seven of 10 patients received GJG before the initiation of GnP treatment. There was no association between the duration of administration of GJG and the severity of ILD (*P* = 0.324).

**Table 4:** Demographics of the 10 patients who developed GnP-induced ILD

	Age	Gender	GnP number of doses	Date of onset	History of lung disease	History of chemotherapy	KL-6 at onset	Treatment	Grade	Concomitant medications	Duration of administration of GJG
1	69	M	11	108	COPD	mFFX 4C	287	mPSL+PSL	3	GJG, RZ	50
2	68	M	28	302	No	No	192	No	1	GJG	134
3	57	F	6	54	No	mFFX 4C	527	No	1	EZ, LP	–
4	79	F	4	43	No	No	3359	No	1	GJG, RZ	43
5	70	M	12	162	No	mFFX 6C	1822	No	1	GJG, RZ	252
6	64	F	6	65	No	No	578	PSL	3	RZ	–
7	72	F	8	80	No	No	6342	mPSL+PSL	3	RZ, LP	–
8	70	M	3	34	No	mFFX 29C	293	mPSL+PSL	2	GJG, RZ	39
9	79	F	8	77	No	No	899	mPSL+PSL	2	GJG, RZ	77
10	65	M	8	139	No	No	409	No	1	GJG, NX, RZ	139

mFFX consists of oxaliplatin 85 mg/m^2^, leucovorin 200 mg/m^2^, irinotecan 150 mg/m^2^, and continuous intravenous infusion of 5-FU at 2400 mg/m^2^, administered every 2 weeks. mPSL was administered at 0.5–1 g/day for 3 days, and PSL was administered at 0.5–1 mg/kg /day and the doses were gradually reduced over the clinical course. GJG was administered at 2.5 g three times daily. RZ was administered at 10 mg once daily. EZ was administered at 20 mg once daily. LP was administered at 60 mg three times daily. NX was administered at 100 mg three times daily

*mFFX* Modified FOLFIRINOX therapy, *mPSL* Methylprednisolone, *PSL* Prednisolone, *GJG* Goshajinkigan, *RZ* Rabeprazole Na, *EZ* Esomeprazole, *LP* Loxoprofen Na, *NX* Naproxen

### Association between GJG preventive administration and CIPN severity

The incidence of CIPN among all cases and the association of CIPN severity with GJG preventive administration were evaluated. Among the patients receiving GnP, 72 (69%) developed CIPN, and the severity was Grade 1 in 41 (39%), Grade 2 in 24 (23%), and Grade 3 in 7 (7%). As the background of patients who be onset CIPN, there was no difference in age, sex, nabPTX cumulative dosage, the pretreatment history of oxaliplatin, and the history of CIPN at the initiation of GnP therapy between with and without GJG. Also, there was no difference in the use of pregabalin between with and without GJG. GJG administration appeared to have no protective effect against development of CIPN (Table 5).

**Table 5:** Association between goshajinkigan preventive administration and CIPN severity

n = 105	GJG (+) *n* = 23	GJG (−) *n* = 82	*P*
Age (years)	70 (57–79)^c^	66 (37–79)^c^	0.535^a^
Gender (male/female)	16 / 7	55 / 27	0.822^b^
nabPTX cumulative dosage (mg)	1112 (400–7364)^c^	1589 (360–7226)^c^	0.139^a^
Pretreatment history of oxaliplatin, n (%)	8 (35%)	35 (43%)	0.496^b^
Oxaliplatin cumulative dosage (mg)	941 (510–4399)^c^	1040 (100–4405)^c^	0.679^a^
History of CIPN at the initiation of GnP, n (%)	9 (39%)	54 (65%)	0.371^b^
Pretreatment history of pregabalin, n (%)	4 (17%)	4 (49%)	0.068^b^
CIPN severity with GnP therapy			
CIPN (−), n (%)	4 (17%)	29 (35%)	0.716^a^
Grade 1, n (%)	12 (52%)	29 (35%)	
Grade 2, n (%)	7 (30%)	17 (21%)	
Grade 3, n (%)	0 (0%)	7 (9%)	

The severity of CIPN was evaluated based on the medical records of medical staff at the time of discontinuation of GnP therapy or at the time of cutoff

*GJG* Goshajinkigan, *CIPN* Chemotherapy-induced peripheral neuropathy

^a^Mann-Whitney *U*-test, ^b^Fisher’s exact test

^c^Median (minimum-maximum)

## Discussion

In this study, we investigated the incidence of ILD in PDAC patients receiving GnP therapy at our hospital. In the phase III international multicenter study of GnP therapy, the frequency of ILD was 4.0% (17/421) and the median period to develop ILD from initiation was 86 days [3]. Similarly, Takeda et al. reported that the incidence of ILD was 2.2% (20/910) in the PDAC patients receiving GnP therapy, and the median time to onset of ILD was 80 days [10]. On the other hand, Irie et al. observed a higher frequency of ILD in a small group of PDAC patients given GnP therapy (18.9%, 7/37), and reported that the severity of ILD was mild, except for one patient, who recovered with steroid therapy [11]. In our study, the incidence of ILD was 9.5% (10/105), and the median period to ILD onset was 79 days. Furthermore, ILD improved in all cases in response to treatment early after onset.

Next, we examined candidate risk factors for ILD and identified the administration of goshajinkigan used for CIPN prevention as a risk factor. GJG is composed of *Achyranthis Radix, Rehmanniae Radix, Dioscoreae Rhizoma, Corni Fructus, Alismatis Rhizoma, Plantaginis Semen, Moutan Cortex, Poria, Processi Aconiti Radix* and *Cinnamomi Cortex*, and is used to treat dysuria, pollakiuria, edema, low back pain, etc. It is often used in the treatment of CIPN based on its putative peripheral blood flow-promoting and antinociception effects [12, 13]. There have been three case reports suggesting that GJG caused ILD [14–16]. Those cases showed no increase in LDH or KL-6, and recovered in response to steroid treatment after discontinuing GJG. In our study, an increase in KL-6 was observed in 6/10 cases, but all recovered with steroid treatment after discontinuation of GJG, in line with the previous reports. We found no relationship between the period of GJG administration and the severity of ILD. The reported period from initiation of Kampo medicines to the appearance of ILD varied from several hours to 1 year [17–19], and it was unclear whether the appearance of ILD is dose-dependent. It was proposed that the induction of ILD by Kampo medicines is due to allergic reactions (mainly type III and type IV) [20], but the mechanism remains to be fully established. There has been no previous report on the onset of ILD caused by the combination of anticancer drugs and Kampo medicines. However, the number of cases of ILD was small in the present study, so further studies are needed to clarify the situation. Interestingly, we found no significant correlation between ILD onset and previously reported risk factors [4–9] in this study, but this may be due to the limited number of cases and the small numbers of comorbidities such as lung cancer and lung disease and poor performance status.

CIPN is a painful side effect for patients, and nabPTX contained in GnP can induce CIPN. Cryotherapy and the use of frozen gloves have been reported as preventive measures for CIPN [21, 22]. Prophylactic administration of GJG has also been suggested, though its effectiveness is unclear [23–26]. In this study, we found no preventive effect of GJG against CIPN. Furthermore, meta-analysis has indicated that GJG does not reduce the severity of CIPN in patients with colorectal cancer and breast cancer [27, 28]. In addition, the CIPN Management Guide of the Cancer Support Society states that administration of GJG is not recommended for the prevention of CIPN symptoms caused by oxaliplatin [29], although taxane drugs including nabPTX are not mentioned. Our results suggest that administration of GJG to prevent CIPN may not be desirable.

Limitations of this study include the small number of cases, reflecting the incidence rate of ILD, and therefore we could not perform multivariate analysis. In addition, unified regular respiratory symptom monitoring could not be performed due to the retrospective nature of the study. According to the Abraxane® Proper Use Guidelines (Taiho Yakuhin Kogyo Co., Ltd., December 2019 Revised), regular interviews with patients about initial symptoms such as fever, cough, shortness of breath, and dyspnea, auscultation, inspections of chest X-rays and CT, and clinical laboratory tests such as KL-6 values are recommended for early detection of ILD during GnP therapy. It will be important to develop a unified evaluation system for early detection of ILD in the hospital. Prospective trials based on the objective evaluation of ILD are also desirable. Also, we cannot rule out the possibility of that ILD was induced by GJG in some patients. At present, the frequency of GJG-induced ILD is unknown and there are only three reports of GJG-induced ILD to date. Finally, we could not confirm patients’ compliance with the prescribed GJG treatment during outpatient visits.

In conclusion, the incidence of ILD in PDAC patients treated with GnP therapy at our hospital was 9.5%, and coadministration of GJG appeared to be a risk factor for ILD. In addition, we found that preventive administration of GJG does not reduce the severity of CIPN. There is still no clear evidence that GJG can prevent CIPN, so GJG administration may need to be reconsidered, and in any event respiratory symptoms should be carefully monitored.

## Data Availability

All data generated or analyzed during this study are included in this published article.
